# Peripheral and central vascular conductance influence on post-exercise hypotension

**DOI:** 10.1186/1880-6805-31-32

**Published:** 2012-12-18

**Authors:** Masako Y Endo, Kazue Shimada, Akira Miura, Yoshiyuki Fukuba

**Affiliations:** 1 Department of Exercise Science & Physiology, School of Health Sciences, Prefectural University of Hiroshima, 1-1-71 Ujina-higashi, Minami-ku, Hiroshima, 734-8558, Japan

**Keywords:** Regional hemodynamics, Central hemodynamics, Post-exercise hypotension, Doppler ultrasonography

## Abstract

**Background:**

Post-exercise hypotension (PEH) following prolonged dynamic exercise arises from increased total vascular conductance (TVC) via skeletal muscle vasodilation. However, arterial vasodilation of skeletal musculatures does not entirely account for the rise in TVC. The aim of the present study was to determine the contribution of vascular conductance (VC) of the legs, arms, kidneys and viscera to TVC during PEH.

**Methods:**

Eight subjects performed a single period of cycling at 60% of heart rate (HR) reserve for 60 minutes. Blood flow in the right renal, superior mesenteric, right brachial and right femoral arteries was measured by Doppler ultrasonography in a supine position before exercise and during recovery. HR and mean arterial pressure (MAP) were measured continuously. MAP decreased significantly from approximately 25 minutes after exercise cessation compared with pre-exercise baseline. TVC significantly increased (approximately 23%; *P* <0.05) after exercise compared with baseline, which resulted from increased VC in the leg (approximately 33%) and arm (approximately 20%), but not in the abdomen.

**Conclusion:**

PEH was not induced by decreased cardiac output, but by increased TVC, two-thirds of the rise in which can be attributed to increased VC in active and inactive limbs.

## Background

In healthy individuals, an acute period of dynamic exercise is known to elicit a reduction in arterial blood pressure relative to pre-exercise levels for approximately two hours [1-3]. This post-exercise hypotension (PEH) produces anti-hypertensive effects relevant to hypertensive patients [2]. In fact, in such patients, the anti-hypertensive effects after exercise can last beyond half a day [1-3]. Thus, it is important to determine the precise mechanisms underlying PEH.

In most individuals, PEH is considered to be caused by an increase in total vascular conductance (TVC) that is incompletely offset by an increase in cardiac output [1-3], although in older hypertensive patients [4] and in endurance-trained men [5], PEH is largely mediated by decreased cardiac output. The increased TVC that causes PEH is predominantly attributed to increased vascular conductance (VC) resulting from vasodilation in exercise and non-exercise limbs [1-3]. However, the results of previous studies have shown only qualitative changes of VC in limbs; thus, it is unclear whether the vascular beds in arm and leg quantitatively fully account for the rise in TVC during PEH. Because we observed that the blood flows and VC in kidney and intestine were reduced during moderate cycling exercise, even during exercise for only four minutes [6], the increased TVC during PEH might be associated with prolonged over-perfusion in these organs after exercise. However, against our expectation, Pricher *et al.*[7] found no direct role of the abdominal circulation in the increased TVC following moderate aerobic exercise. Their methods to determine splanchnic and renal blood flow (BF) were dye dilution and p-aminohippuric acid clearance. These methods are invasive techniques with physical suffering and do not allow the BFs in multiple blood vessels (such as those supplying different skeletal muscles) to be measured simultaneously. More recently, Doppler ultrasonography has been used as a noninvasive method for direct measurement of flow velocity in several vessels [8-10]. This noninvasive method has caused little physical stress to the subjects compared with the previous invasive method [7], therefore, it enables to exclude the effect of such stress on the hemodynamics responses during PEH.

Previous studies reported that the rise in TVC during the hypotension after cycling exercise was caused by the increase of VC in exercised (that is, leg) and non-exercised (that is, arm) limbs. However, because the ratio of blood flow in the arms to the total blood flow was much smaller than that in the legs, we hypothesized that the increased VC in the arms may contribute little quantitatively to the increased TVC during PEH. Therefore, the quantitative contribution of regional VC to the rise in TVC from the viewpoint of ‘whole body coordination’ during PEH needs to be explained. To our knowledge, there are no human studies investigating the vascular responses of several organs and tissues simultaneously during PEH using pulsed-Doppler ultrasonography.

In the present study, to explain which vascular beds in regional organs and tissues contributed quantitatively to the increased TVC during PEH, we examined the VC in leg, forearm, viscera and kidneys using pulsed-Doppler ultrasonography.

## Methods

### Subjects

Twelve healthy, normotensive, non-smoking volunteers (nine males, three females) participated in the present study and gave written informed consent. The study was approved by a local ethics committee and was in accordance with the Declaration of Helsinki. The subjects were sedentary with normal activity; they performed no regular endurance training and participated in <2 h of aerobic exercise per week. Each subject underwent a pilot examination before performing the main protocol. During the pilot examination, we attempted to measure the mean arterial velocities (MBVs) of renal and superior mesenteric arteries from high-quality ultrasound Doppler signals with B-mode images. However, this was not feasible in four subjects owing to abdominal gas and the difficulty in obtaining vessel images at spontaneous end-expiration. Therefore, eight subjects (five men, three females) participated in the main protocol. Subjects were aged 20 to 32 years, and had a mean ± SE height and weight of 169 ± 4 cm and 60 ± 3 kg, respectively.

The subjects arrived in the laboratory after having abstained from caffeine and exercise for at least one day, and from food and drink for at least three hours. Each subject underwent an incremental cycling exercise test (232CXL; Combi, Tokyo, Japan) to determine the work rate for the main protocol. Subjects performed continuous graded cycle ergometer exercise at 30, 60 and 90 W each for 5 minutes at 60 rpm. Heart rate (HR) was monitored via a three-lead electrocardiogram (ECG) (BP-88S; Colin, Tokyo, Japan) during this incremental cycling exercise. To determine the work rate for the main protocol, the linear relationship between steady-state HR at the end of each stage and work rate was calculated, and the work rate corresponding to HR at 60% of resting HR (RHR) reserve (calculated as 0.6*((220-age)-RHR)+RHR) was estimated. This work-load was used for the 60-minute exercise period. The room temperature was maintained at 24 ± 1°C by a thermal feedback device.

### Protocol

Subjects were positioned supine for 40 minutes before exercise. The exercise consisted of a 60-minute period of upright cycling at 60 rpm. Immediately after the exercise period, the subjects were positioned supine for a further 60 minutes. The central and peripheral arterial hemodynamic parameters were obtained during the periods 25 to 40 minutes prior to exercise (pre), and 15 to 30 minutes (post 1) and 40 to 55 minutes (post 2) after exercise.

### Measurements

HR was continuously monitored via a three-lead ECG throughout the protocol. BP (blood pressure) was measured using an autonomic manometer (BP-306; Colin, Tokyo, Japan) with a left arm cuff, every five minutes during pre- and post-exercise. BP during exercise was measured using a mercury manometer (601MY; Kenzmedico, Saitama, Japan) at 20 and 50 minutes after the start of exercise with the left arm at heart level.

Cardiac output (CO) was determined using an echocardiograph (EUB6500; Hitachi Medical Corporation, Tokyo, Japan). To obtain a parasternal long-axis view, a 2.5-MHz annular phased-array sector probe was placed at the point of maximal cardiac impulse on the chest wall in an intercostal space between the third and fourth ribs. The beat-by-beat left ventricular volumes were measured by one investigator for at least 10 s (6 to 9 beats). M-mode and two-dimensional B-mode images were stored simultaneously to the hard disk in the EUB6500, and then saved onto DVD for off-line analysis. The R-R intervals, end-diastolic diameters (EDDs), and end-systolic diameters (ESDs) were analyzed using image analysis software (Image J; National Institutes of Health, Bethesda, Maryland, USA) on a PC. The left ventricular ejection fraction was calculated using Pombo’s formula, where end-diastolic volume (EDV) = π/3*EDD^3^ and end-systolic volume (ESV) = π/3*ESD^3^. Stroke volume (SV) was estimated by EDV-ESV. CO was calculated by SV*HR (calculated by measuring the R-R interval). TVC was calculated from CO/mean arterial pressure (MAP). However, one subject could not analyze the EDD and ESD in post 1, and another subject could not analyze them in post 2 because we could not obtain their M-mode images with high-quality during the measurement. Therefore, we finally analyzed them with one missing value of SV, CO and TVC in post 1 and post 2, respectively.

The BFs of the renal artery (RA), superior mesenteric artery (SMA), right brachial artery (BA), and right femoral artery (FA) were obtained using simultaneous pulsed and echo Doppler ultrasound (LOGIQS6; GE Healthcare Japan, Tokyo, Japan) to measure MBV and arterial blood vessel diameter. The MBV of each vessel was obtained on a beat-by-beat basis using a convex 3.3-MHz probe (for the RA and SMA) and a linear 5.0-MHz probe (for BA and FA) with an insonation angle of 45 to 60°. The diameter of each vessel was measured simultaneously with an imaging frequency of 5.5 MHz (for the RA and SMA) and 12.0 MHz (for the BA and FA). The MBVs in the SMA and RA were measured during breath holding at the spontaneous end-expiration for 20 seconds [10] using the anterior abdominal approach, as previously described [8]. The BA was measured at the level of the antecubital fossa of the right elbow. The FA was measured at a site approximately 2 to 3 cm distal to the inguinal ligament of the right thigh. Each BF was calculated from the MBV and cross-sectional area of each vessel. These BFs were always measured in the order RA, SMA, BA and FA. As a result, we excluded the BF data in the arm of one subject because his data were clearly different from the time-serial change compared with the data of other subjects. Skin BF was measured from the index fingertip of the right arm by laser-Doppler flowmetry (ALF21; Advance, Tokyo, Japan) throughout pre- and post-exercise. The VC of each vessel was calculated as the ratio of each BF to MAP. The BF and VC in skin were calculated as relative to the pre-exercise baseline. The contribution of each regional VC (that is, RA, SMA, BA or FA) to the increased TVC during PEH was calculated by the following equation.

$$\mathrm{Contribution}(\%)=[\frac{\left({\mathrm{VC}}_{\left(\mathrm{post}\right)}–{\mathrm{VC}}_{\left(\mathrm{pre}\right)}\right)}{\left({\mathrm{TVC}}_{\left(\mathrm{post}\right)}-{\mathrm{TVC}}_{\left(\mathrm{pre}\right)}\right)}]*100,$$

where the ‘post’ and ‘pre’ denote the values of post 1 or post 2 during recovery, and of pre-exercise, respectively.

### Statistical analysis

Values are expressed as means ± SE. To test the time-serial changes in each variable, the effect of time on the variables was examined by repeated-measures ANOVA. When a significant F-value was detected, this was further examined by Dunnett’s *post hoc* test against the pre-exercise value. Statistical significance was accepted at *P* <0.05 (SPSS 12.0 for Windows, IBM, Tokyo, Japan).

## Results

### Heart rate and blood pressure response during exercise

HR significantly increased during exercise from 55 ± 3 to 135 ± 3 bpm (at 50 minutes). Systolic blood pressure (SBP) and MAP significantly increased during exercise compared with pre-exercise levels, whereas diastolic blood pressure (DBP) during exercise did not change from the pre-exercise level (pre: SBP 114 ± 4, MAP 78 ± 2, DBP 60 ± 2 mmHg; at 50 minutes of exercise: SBP 147 ± 5, MAP 87 ± 2, DBP 59 ± 3 mmHg).

### Central and regional hemodynamics responses during PEH

**Figure 1:**
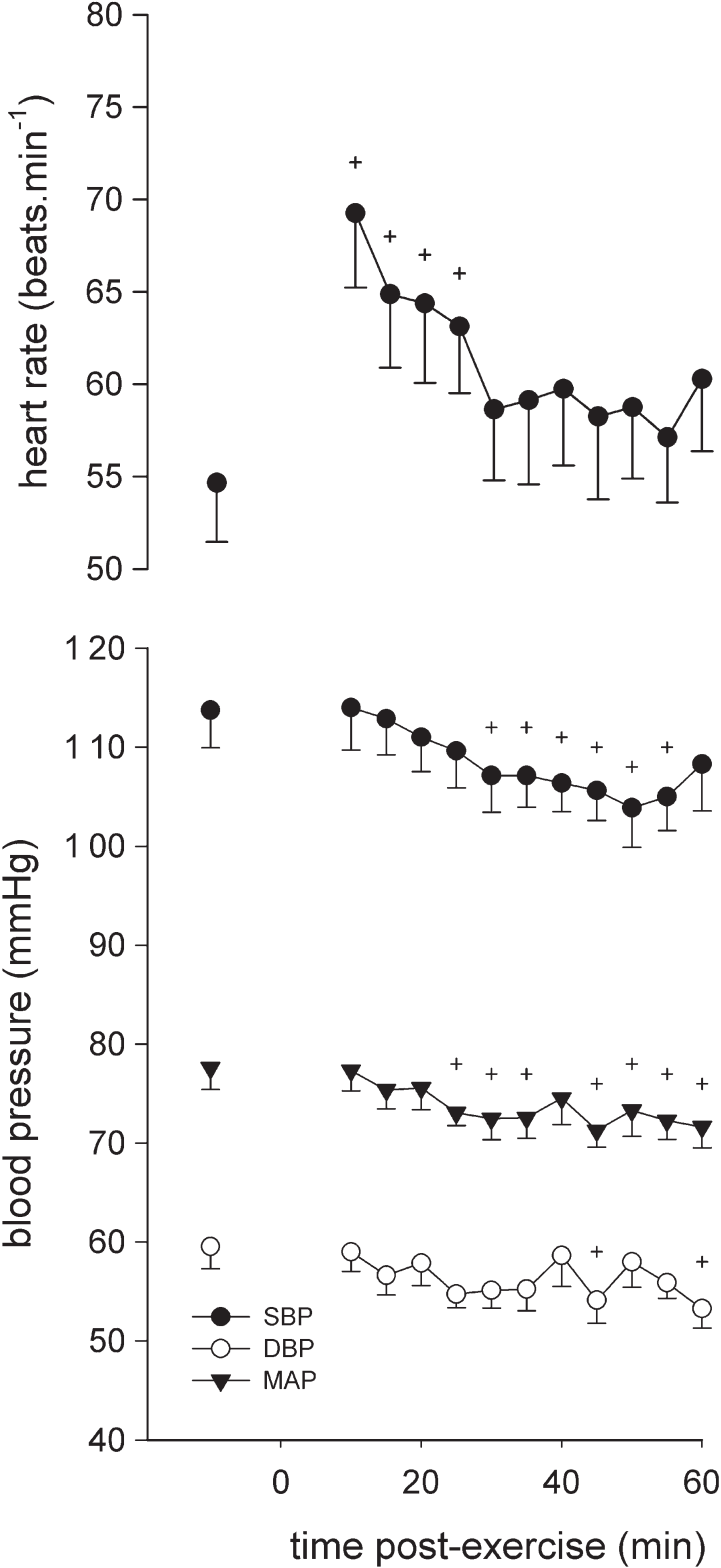
**Heart rate (top) and blood pressure (bottom) before (left) and after exercise.** Circles, triangles and squares show systolic (SBP), mean (MAP) and diastolic (DBP) blood pressure data, respectively. Values are means ± SE. + significantly different from the pre-exercise value (*P* <0.05).

The time courses of HR and blood pressure responses during post-exercise are shown in Figure 1. HR increased until approximately 25 minutes after the cessation of exercise, and then recovered. SBP and MAP consistently decreased in all subjects from approximately 30 minutes and 25 minutes, respectively, after exercise cessation, whereas DBP remained almost constant throughout the recovery. 

**Figure 2:**
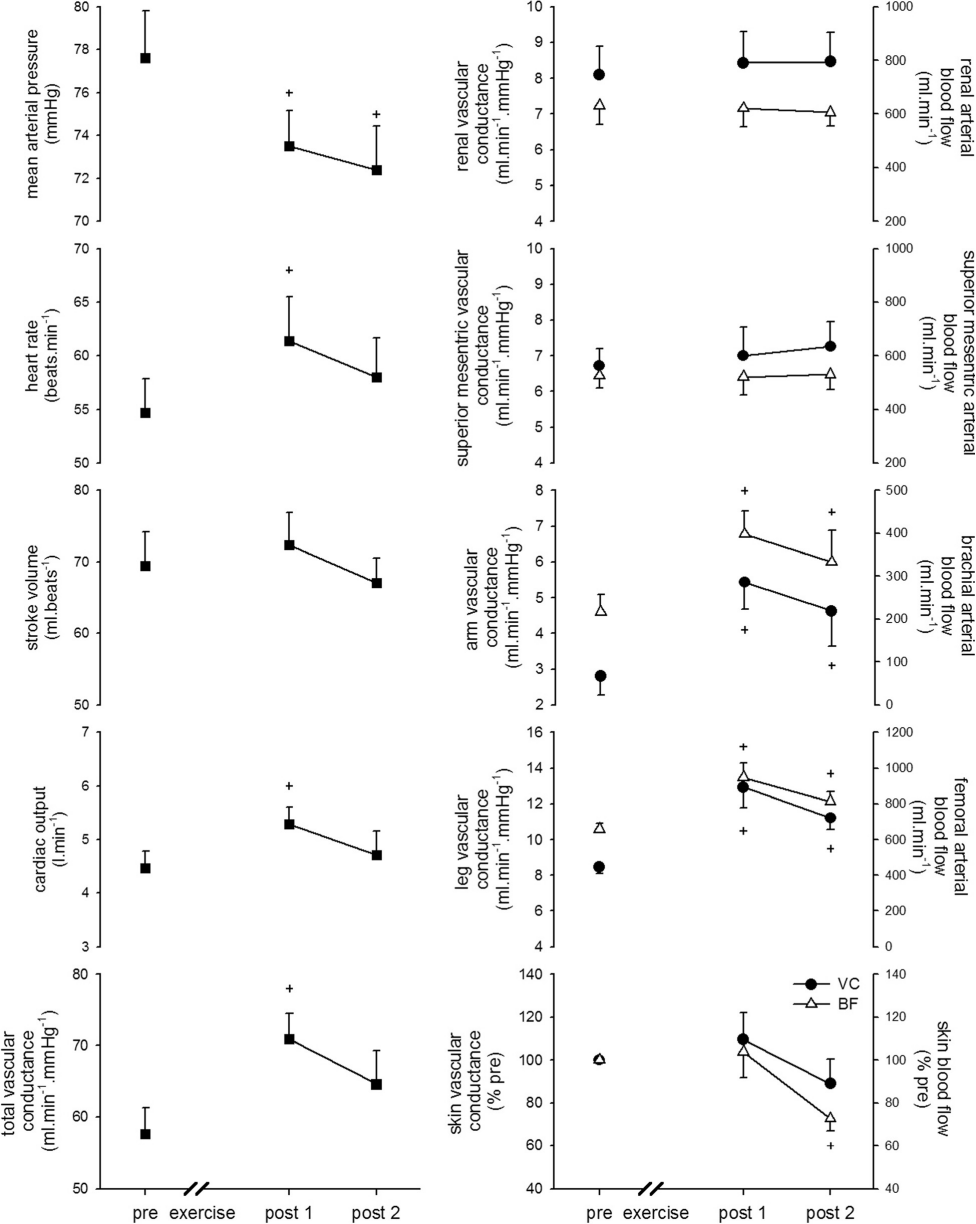
**Systemic (left) and regional (right) hemodynamics before (pre) and after (post 1 and 2) exercise.** Circles and triangles show the vascular conductance (VC) and blood flow (BF) data, respectively. Values are means ± SE. + significantly different from the pre-exercise value (*P* <0.05).

Central and regional hemodynamic responses of pre- and post-exercise can be seen in Figure 2. MAP significantly decreased at both post 1 and post 2 during recovery (pre: 77.6 ± 2.2; post 1: 73.5 ± 1.7; post 2: 72.4 ± 2.1 mmHg). CO was significantly elevated at post 1 (pre: 4.47 ± 0.32; post 1: 5.28 ± 0.33; post 2: 4.71 ± 0.45 l/min). As a result, TVC was significantly increased at post 1 (pre: 57.6 ± 3.7; post 1: 70.9 ± 3.6; post 2: 64.6 ± 4.7 ml/min/mmHg). The increase in TVC paralleled the increase in VC in the leg and arm. However, the VC of the RA and SMA did not change from pre-exercise levels. Skin VC tended to decrease toward the end of recovery. The response of each BF was similar to the response of each VC. 

### Contribution of regional VC to the increased TVC during PEH

TVC increased by approximately 23% and 12% at post 1 and post 2, respectively, compared with the pre-exercise values. The rise in TVC at post 1 resulted from increased VC in the legs (approximately 33%), arms (approximately 20%), kidneys and viscera (approximately 2%), and other organs and/or tissues (approximately 43%). The rise in TVC at post 2 resulted from increased VC in the legs (approximately 39%), arms (approximately 26%), kidneys (approximately 5%), viscera (approximately 8%), and other organs and/or tissues (approximately 22%).

## Discussion

The purpose of this study was to clarify which vascular beds in regional organs and tissues contribute to the increased TVC during PEH using ultrasound Doppler. The major findings of the present study were as follows. 1) the contributions of VC in the legs, arms, kidneys and viscera to the increase in TVC during PEH were approximately 33 to 39%, 20 to 26%, 2 to 5% and 2 to 8%, respectively; therefore, the VC of active and non-active limbs accounted for approximately two-thirds of the rise in TVC during PEH. 2) The VC of skin in the fingertip was unchanged after exercise. These data suggest that the arterial vasodilation in active and inactive limbs, but not in the kidney, viscera and/or skin in the fingertip explains a large part of the rise in TVC during PEH.

In the majority of individuals, it is well known that PEH is caused by an increase in TVC that is not fully offset by a rise in CO [1-3]. This increase in TVC is thought to predominantly arise from increased VC following vasodilation in exercised and non-exercised limbs [1-3]. However, those studies only showed a qualitative change in leg and arm vascular beds. In this study, we evaluated a quantitative contribution of several organs and tissues to the rise in TVC after exercise. Previous studies using invasive methods for the measurement of renal and splanchnic BF reported that the VC in splanchnic and renal arteries did not change after moderate cycling exercise [7], and that the decreased renal BF during prolonged exercise returned to pre-exercise levels within 30 minutes after exercise cessation [11]. These data support our finding that the renal and splanchnic vascular beds may not play a direct role in PEH.

As one of our novel findings, this study found that VC in the arms (that is, inactive limbs) makes a certain contribution (namely, 20 to 26%) to the elevated TVC during PEH. It was very intriguing that vasodilation in non-exercise limbs contributed such a large amount to PEH. It may be speculated that the arms were active by gripping the handlebars of the cycle ergometer during exercise. However, this seems to be unlikely, because the work rate in the present study was selected as moderate intensity, not severe and/or maximal intensity, and all subjects put their relaxed arms on the handlebars during cycling exercise for 60 minutes. Therefore, vasodilation in the arms during PEH may be induced by an increase in cutaneous circulation for the regulation of body temperature.

In the present study, approximately 22 to 43% of the TVC rise during PEH remains to be accounted for. Although we measured glabrous skin BF in the fingertip using laser-Doppler flowmetry, we could not quantitatively calculate its contribution to the TVC rise. In addition, unfortunately, we could not measure the cutaneous circulation at several skin sites during PEH. The blood flow to limbs were affected by the vascular response in skeletal muscles, skin and other tissues; therefore, the increased VC in arms and legs during PEH might be caused by not only the vasodilation in skeletal muscles but also a sustained vasodilation in cutaneous vasculature at post-exercise. Contrary to our expectation, Willkins *et al.*[12] reported that the time serial changes in cutaneous VC at four non-glabrous skin sites (chest, forearm, thigh and leg) did not parallel the reduction in arterial pressure after exercise; therefore, they concluded the cutaneous VC did not play an obligatory role in PEH. However, observing the time course of cutaneous VC in the early phase of post-exercise (particularly, in the phase corresponding to post 1 in the present study), the cutaneous VC in the thigh and chest remained increased compared with the pre-exercise level; hence, the contribution of skin VC may not be negligible in the early phase during PEH. In any case, further studies are required to determine the quantitative contribution of other possible vascular beds, such as brain, heart and/or skin to the rise in TVC during PEH.

In this study, we did not determine the mechanism underlying the differential vascular response in each organ and tissue during PEH, but this difference was postulated to be related to the differential outflow of sympathetic vasoconstrictor nerve activity and the attenuation of vascular responses to sympathetic vasoconstrictor outflow via impaired α adrenergic receptor stimulation by local vasodilator substances. Muscle sympathetic nerve activity in borderline hypertensive subjects [13] and normotensive subjects [14] is decreased after exercise compared with pre-exercise levels. In an animal model of hypertension, differential sympathetic outflow to a few organs and tissues was observed during the reduced arterial pressure following prolonged treadmill exercise [15] and/or prolonged stimulation of the sciatic nerve [16]. The differential vascular response at each arterial smooth muscle may be regulated as a result of the competing influences of local vasodilator substances and modulation of the α adrenergic pathway. The kidneys, spleen and skin are highly sensitive to α adrenergic receptor stimulation, whereas skeletal and cardiac muscles are not [3]. The relationship between increased nitric oxide or histamine and the TVC change after exercise have been reported in humans [17,18]; however, the neural and vascular responses during PEH remain unknown.

## Conclusion

In conclusion, our results demonstrated that approximately two-thirds of the rise in TVC during PEH is induced by arterial vasodilation in active and inactive limbs, but not in the renal and splanchnic vascular beds. However, it remains unclear which other vascular beds affect one-third of the TVC rise during PEH. Future studies are required to investigate simultaneously how other possible vascular beds, such as the brain, heart and skin, contribute to the rise in TVC during PEH.

## Abbreviations

ANOVA: analysis of variance; BA: brachial artery; BF: blood flow; BP: blood pressure; CO: cardiac output; DBP: diastolic blood pressure; ECG: electrocardiogram; EDD: end-diastolic diameter; ESD: end-systolic diameter; FA: femoral artery; HR: heart rate; MAP: mean arterial pressure; MBV: mean arterial velocity; PEH: post-exercise hypotension; RA: renal artery; RHR: resting heart rate; SBP: systolic blood pressure; SE: standard error; SMA: superior mesenteric artery; SV: stroke volume; TVC: total vascular conductance; VC: vascular conductance.

## Competing interests

The authors declare that they have no competing interests.

## Authors’ contributions

MYE contributed to the conception and design of the study, performed the experiment, and drafted the manuscript. KS contributed to data collection and analysis. AM contributed to the data interpretations. YF contributed to modify draft by intellectual content. All authors read and approved the final manuscript.
